# Evaluation of Daughter Radionuclide Release from the ^103^Pd/^103m^Rh In Vivo Generator for Targeted Auger Therapy

**DOI:** 10.3390/ph19010126

**Published:** 2026-01-11

**Authors:** Aicha Nour Laouameria, Cathryn H. S. Driver, Monika Buys, Elena Sergeevna Kurakina, Mátyás Hunyadi, Jan Rijn Zeevaart, Zoltan Szucs

**Affiliations:** 1 Doctoral School of Chemistry, University of Debrecen, Egyetem tér 1, 4032 Debrecen, Hungary; laouameria.aicha.nour@atomki.hu; 2 HUN-REN Institute for Nuclear Research, Bem tér 18/c, 4026 Debrecen, Hungary; hunyadi@atomki.hu (M.H.); zszucs@atomki.hu (Z.S.); 3 NuMeRI, Nuclear Medicine Research Infrastructure NPC, Steve Biko Academic Hospital, Pretoria 0084, South Africa; cathryn.driver@necsa.co.za; 4 Applied Radiation, South African Nuclear Energy Corporation (Necsa), Pelindaba 0240, South Africa; monika.buys@necsa.co.za; 5 Dzhelepov Laboratory of Nuclear Problems, Joint Institute for Nuclear Research, Dubna 141980, Russia; kurakina@jinr.ru

**Keywords:** ^103^Pd/^103m^Rh in vivo generator, Auger electron emitters, daughter radionuclide release, recoil effect, DOTA-TATE, phthalocyanine-TATE, DOTA-TOC

## Abstract

**Background/Objectives**: The ^103^Pd/^103m^Rh in vivo generator represents a promising Auger electron-emitting system, in which both parent and daughter radionuclides emit predominantly Auger electrons with minimal accompanying radiation. This study investigates the release dynamics of daughter radionuclides from the ^103^Pd/^103m^Rh in vivo generator and evaluates the underlying mechanisms governing bond rupture and daughter retention. **Methods**: Cyclotron irradiation of rhodium foils was performed in two separate batches, followed by radionuclide separation using conventional wet chemistry and a novel dry distillation technique. The purified ^103^Pd radionuclide was used to radiolabel DOTA-TATE, phthalocyanine-TATE, and DOTA-TOC chelators. The resulting complexes were immobilized on Strata-X and Strata-C18 solid-phase extraction columns. Scheduled elution experiments were conducted to quantify the release of the ^103m^Rh daughter radionuclide. **Results**: The measured ^103m^Rh release rates were 9.8 ± 3.0% and 9.6 ± 2.7% from Strata-X columns with DOTA-TATE and phthalocyanine-TATE, respectively, and 10.5 ± 2.7% and 12.0 ± 0.5% from Strata-X and Strata-C18 columns, respectively, with DOTA-TOC. These values are significantly lower than the ~100% release predicted based on the reported Auger electron yield of 186%. One explanation for this difference could be potential inconsistencies in decay data that may require correction; this needs further investigation. The results further demonstrated that delocalized π-electrons, introduced via phthalocyanine-based chelation, did not mitigate daughter release. **Conclusions**: The low observed daughter nuclide release represents a favorable characteristic for the future clinical translation of the ^103^Pd/^103m^Rh Auger emitter pair. The findings support the conclusion that Auger electron cascades, rather than nuclear recoil energy, dominate bond rupture processes.

## 1. Introduction

**Figure 1:**
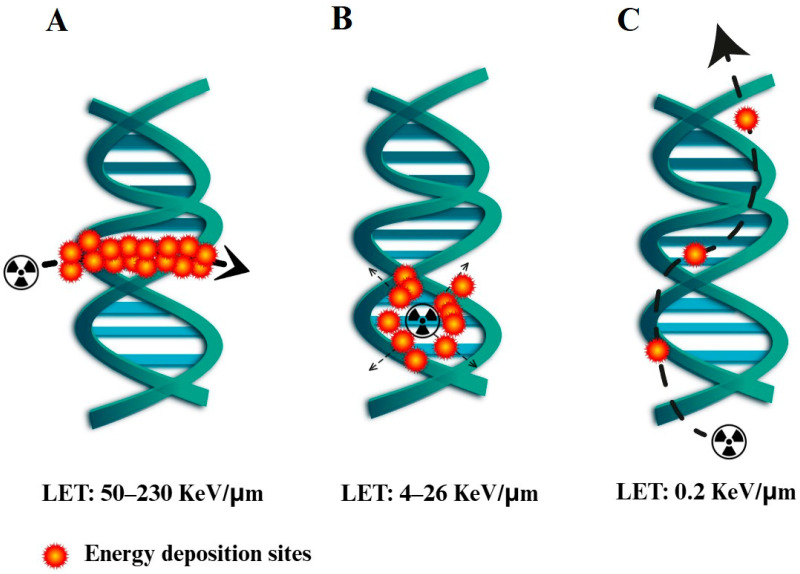
Comparison of radiation emissions: (**A**) alpha particles, (**B**) Auger electrons, and (**C**) beta particles. LET: linear energy transfer [8].

Targeted radionuclide therapy (TRNT) offers an advanced approach in cancer treatment by localizing cytotoxic radiation to tumor tissues with high specificity [1]. While beta(β^−^)-emitters and alpha(α)-emitters have shown effectiveness, their tissue penetration spans micrometers to millimeters, raising concerns about collateral damage [2]. Alternative radionuclides being investigated for TRNT include Auger electron emitters, as the tissue penetration of Auger electrons ranges from nanometer to micrometer scales [3]. The energy released by β^−^-emitting radionuclides is typically sufficient only to induce single-strand DNA breaks, whereas both α-particles and Auger electrons are capable of producing double-strand breaks (DSBs) [4,5]. Cells are generally able to recognize and repair single-strand breaks [6], but DSBs represent a critical form of damage that often leads to apoptosis or irreversible loss of viability [7]. Similarly to nuclear DNA damage, high-dose irradiation of mitochondria is also lethal for the cell, and this effect can only be achieved by radiation with sufficiently high linear energy transfer (LET). Alpha particles exhibit the highest LET (50–230 keV/µm), while Auger electrons fall into an intermediate LET range (4–26 keV/µm), significantly higher than that of beta particles (≈0.2 keV/µm) (Figure 1) [8].

Despite not reaching the LET values of α-emitters, an important advantage of Auger electron emitters over α-emitting radionuclides for tumor therapy is their extremely short penetration range, which makes them uniquely capable of inducing double-strand DNA breaks (DSBs) while sparing adjacent healthy tissue. For instance, simulations using Geant4-DNA have demonstrated that Auger electron emitters can produce multiple DSBs, underscoring their potential in targeted cancer therapy [9]. These results substantiate that Auger therapy can effect potent subcellular damage, supporting its therapeutic potential. In contrast, α-particles travel across several cell diameters, thereby exposing adjacent healthy tissue to collateral damage.

In addition, a major drawback of α-emitters arises from their decay properties: the daughter radionuclide may be ejected from its chelator due to the substantial recoil energy imparted by the emitted α-particle. This recoil can disrupt metal–ligand coordination bonds, releasing free radioactive daughters into circulation. A well-documented example is Actinium-225 (^225^Ac), in which the recoil-induced release of daughter radionuclides resulted in uncontrolled irradiation and toxicity in off-target tissues [10]. This mechanism, traditionally referred to as the Szilard–Chalmers effect, encompasses both recoil-induced displacement and the chemical instability following nuclear transformation [11]. This release of the daughter radionuclide from the chelator (Francium-221 (^221^Fr) in the case of Actinium-225 (^225^Ac)) means that the chemistry changes from actinide to an alkali metal ion that is a potassium analog and is likely to be displaced out of the cell as part of the cell’s Na/K pump, which can lead to the relocation of the daughter radionuclide to surrounding tissue. Although (^221^Fr) has a short half-life of 4 min, it is postulated to be responsible for some off-target irradiation experienced with (^225^Ac)-based compounds [10]. However, this phenomenon is not unique to (^225^Ac), and all vivo generators have the same risk, including Auger emitters.

The theoretical radiation risk associated with recoil effects in Auger electron emitters was previously evaluated by our group for the Palladium-103/Rhodium-103m (^103^Pd/^103m^Rh) parent–daughter system [12]. ^103^Pd (t_1/2_ = 16.99 days) decays by electron capture, releasing Auger electrons and X-rays (20–23 keV), to ^103m^Rh (t_1/2_ = 56.1 min), which undergoes isomeric transition to stable ^103^Rh with the emission of additional Auger electrons and low-intensity gamma (γ)-rays [13]. Based on mathematical estimations derived from the Standard Model, the recoil energy from the ^103^Pd/^103m^Rh–Auger transitions was considered insufficient to break the chelator–metal bond, suggesting that daughter radionuclides should remain stably coordinated. The Standard Model predicts that, for a mass of 100, the limiting neutrino or photon energy will be 0.75 MeV [14], whereas the Q-value for ^103^Pd decay is only 0.543 MeV. However, further experimental studies conducted using the Dysprosium-166/Holmium-166 (^166^Dy/^166^Ho) system contradicted this prediction, demonstrating that a measurable fraction of daughter radionuclides are indeed released and appear as free ions [15]. This discrepancy led to the hypothesis that the proportion of free daughter ions is very likely correlated with the number of Auger electrons emitted [16], a relationship that was supported by previous independent studies [17].

In subsequent work, our group postulated that daughter release from the chelator is driven by the fragmentation of the chelating ring due to an acute local electron deficit following the Auger emission process [18]. It is well established that an atom undergoing decay with subsequent Auger cascades loses a substantial number of electrons. For example, the decay of ^125^I results in the formation of ^125^Te^21+^, corresponding to the ejection of 21 electrons from the atom. Such extreme electron loss results in an ‘electron sink’, drawing in electrons from the immediate chemical environment in order to compensate for the deficit. These electrons would most likely originate from nearby binding orbitals, including those of the chelator coordinating the metal ion, or, in the case of ^125^I-labeled nucleotides such as deoxyuridine, from the DNA itself. Nath et al. [19] proposed that delocalized, π-electron-rich chelators may mitigate such fragmentation by supplying electrons to buffer the local deficit. Experimental evidence for this hypothesis was demonstrated with phthalocyanine in the case of ^57^Co decaying to ^57^Fe via electron capture, where the presence of the delocalized π-system reduced the dissociation of ^57^Fe from the phthalocyanine chelator. Based on this rationale, phthalocyanine-TATE is included in the present study to evaluate whether delocalized π-electrons could reduce fragmentation compared to DOTA-TATE and DOTA-TOC.

In this study, we investigate the experimental measurement of free ion release in the Palladium-103/Rhodium-103m (^103^Pd/^103m^Rh) parent–daughter system. This is of relevance since both radionuclides are pure Auger electron emitters and offer the potential for a dual therapeutic effect if ^103^Pd is used in targeted applications, provided that the daughter radionuclide remains confined to the target site defined by the vector molecule. Such radionuclide pairs, termed in vivo generators, are considered highly promising for targeted radionuclide therapy [20].

In terms of clinically relevant chelators, DOTA is the most widely used due to its ability to stably complex a broad range of radionuclides. In peptide receptor radionuclide therapy (PRRT), somatostatin receptor-targeting bioconjugates such as DOTA-TATE and DOTA-TOC are routinely radiolabeled with Gallium-68 for diagnosis and Lutetium-177 for therapy in neuroendocrine tumors [21,22]. These DOTA-TATE/TOC chelator–peptides provide a strong starting point to evaluate daughter release in an in vivo generator system, as such investigations have not yet been systematically performed.

Therefore, using DOTA-TATE, DOTA-TOC, and an additional π-electron-delocalized chelator, phthalocyanine-TATE, as model systems, we quantify the release of the ^103m^Rh daughter radionuclide from the ^103^Pd–chelator system and examine the correlation between the percentage of free ion release and Auger electron emission. In addition, we assess the associated biological risks of daughter release, considering the decay mode, half-life, cellular transport mechanisms, and the broader implications of localized versus delocalized irradiation.

## 2. Results

### 2.1. Optimization of ^103^Pd Production

**Figure 2:**
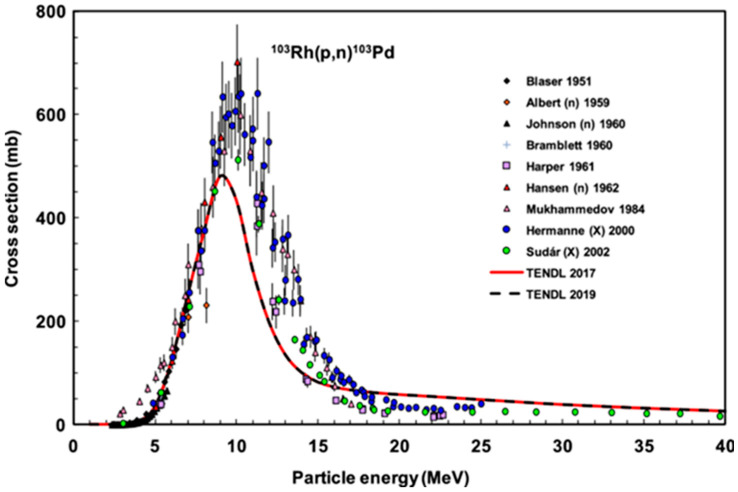
Comprehensive presentation of experimental data and TENDL predictions for the ^103^Rh(p,n)^103^Pd reaction (printed with permission from [23]).

Cyclotron irradiation of the rhodium foil with an 11 MeV proton beam at a current of 20 µA for 30 h produced approximately 6 GBq of ^103^Pd activity, decay-corrected to the end of bombardment (EOB). The activity transferred to Necsa was measured at approximately 100 MBq immediately prior to shipment. The choice of 11 MeV proton energy was deliberate, seeking to minimize side nuclear reactions such as ^nat^Rh(p,pn)^102,102m^Rh and ^nat^Rh(p,p2n)^101,101m^Rh, which would otherwise generate contaminating ^101m^Rh, ^101^Rh, ^102m^Rh, and ^102^Rh isotopes. This optimization is supported by the excitation function data published by Tárkányi et al. [23] (Figure 2), which demonstrate that operation at this energy effectively reduces undesired radionuclidic impurities.

To verify the radionuclidic purity of the produced ^103^Pd and to confirm the minimization of side nuclear reactions at a proton energy of 11 MeV, post-irradiation γ-spectral analysis was performed using HPGe γ-spectroscopy. The detected γ-lines were evaluated against known decay schemes of ^103^Pd/^103m^Rh and potential rhodium contaminants. The identified γ-emissions and their corresponding intensities are summarized in Table 1.

### 2.2. Efficiency of Rhodium–Palladium Separation and ^103^Pd Recovery

The electrochemical dissolution of rhodium foil (as described in Section 4.2 along with safety precautions) proved suboptimal, as it generated hazardous HCl gas, had very slow dissolution kinetics, and resulted in variable ^103^Pd activity recovery. Following anion-exchange chromatography to efficiently separate rhodium and palladium ions, along with trace metal contaminants such as iron (Fe), copper (Cu), zinc (Zn), and cobalt (Co) present at ppb levels in the hydrochloric acid, the recovery of ^103^Pd activity varied between 58% and 85%. The variable yields across dissolution trials were most likely due to poor electrical contact between the Rh foil and graphite electrode once the disintegration of the foil started.

The separation of ^103^Pd ions from the second batch of irradiated ^103^Rh foils using the radionuclide separation equipment (RSE) achieved yields ranging from 72% to 91%. These results validate the efficiency of the diffusion-driven extraction and dry distillation method, which offers an effective alternative to the conventional wet chemistry approach [24] used for the first batch, which resulted in lower ^103^Pd yields and losses of activity. The recovery of ^103^Pd activity from the substrate, following acidic treatment and subsequent purification, achieved rates of 81–83%, further validating this recovery strategy.

### 2.3. Elution of ^103^Pd Complexes and Evaluation of ^103m^Rh Daughter Release

The Strata-X column, featuring a reverse-phase polymeric sorbent, was employed to immobilize the [^103^Pd]Pd–DOTA-TATE complex and the [^103^Pd]Pd–phthalocyanine-TATE complex for recoil effect studies of ^103m^Rh radionuclide release. Additional Strata-X and Strata-C18 columns were used for the immobilization of the [^103^Pd]Pd–DOTA-TOC complex. Three Strata-X columns were used for the [^103^Pd]Pd–phthalocyanine-TATE complex, with five elutions performed for each column (n = 5). Five Strata-X columns were used for the [^103^Pd]Pd–DOTA-TATE complex, with five elutions performed for each column (n = 5). Both the [^103^Pd]Pd–phthalocyanine-TATE and [^103^Pd]Pd–DOTA-TATE complexes were eluted at 5 h intervals over a 20 h period. All eluates were promptly analyzed by gamma spectroscopy.

With a half-life of 56 min, ^103m^Rh radionuclide reaches secular equilibrium with its ^103^Pd parent radionuclide after approximately five half-lives; thus, maintaining a consistent 5 h interval between elutions ensured equilibrium conditions [25].

**Figure 3:**
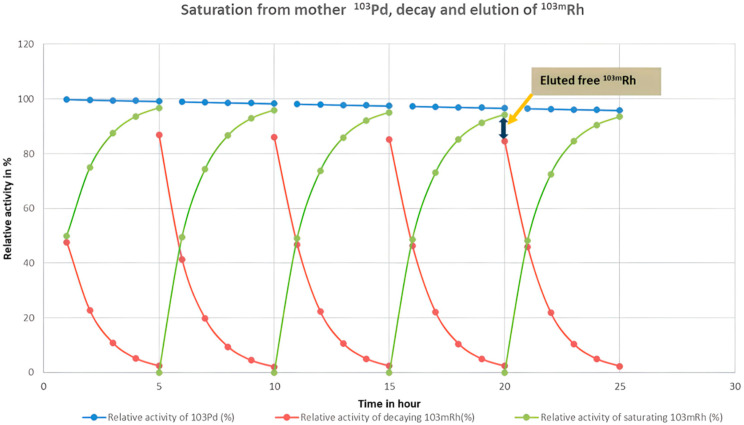
Relative activity ratio of ^103^Pd and ^103m^Rh retained on the Strata-X column over the course of the experiment.

Additionally, maintaining a 5 h half-life interval between elutions ensured the complete decay of any residual ^103m^Rh ions remaining on the column from the previous washing step, thereby standardizing the experimental conditions across all measurements (Figure 3). This figure shows the rate of activity of ^103m^Rh in relation to the decay and the parallel activity generated by the mother ^103^Pd. The chosen 5 h half-life demonstrates an optimal time scale with the generated ^103m^Rm equaling 97% of the theoretical value, as well as the remaining activity from the earlier cycle decays, which is only 3% of the total expected activity.

**Figure 4:**
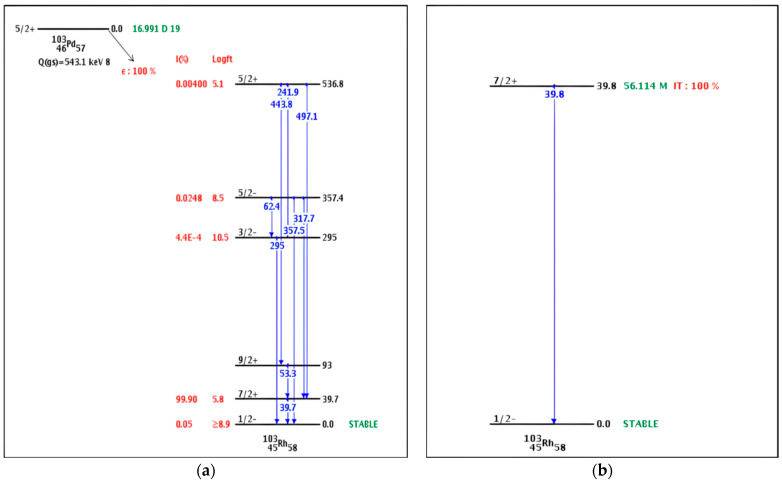
Decay schemes of (**a**) ^103^Pd radionuclide; (**b**) ^103m^Rh radionuclide, showing the transition of ^103^Pd to ^103m^Rh and subsequent decay to stable ^103^Rh, along with associated gamma emissions [26].

The decay schemes of ^103^Pd and ^103m^Rh (Figure 4) show that 99.9% of ^103^Pd decays to ^103m^Rh at an excitation energy of 39.76 keV, and 100% of ^103m^Rh subsequently decays to stable ^103^Rh with the emission of a 39.76 keV gamma photon (intensity: 0.068%). Thus, the difference in measured activity on the column before and after each washing step accurately represents the fraction (1/0.999) of ^103m^Rh released into the eluate. A minor decrease in the intensity of the 357.5 keV gamma peak indicated the minor leakage of ^103^Pd from the chromatographic column; however, this contribution was neglected in the ^103m^Rh activity calculations because measurements were conducted promptly, and any ^103m^Rh generated from eluted ^103^Pd during the measurement period contributed less than ~5% of the total activity measured.

The experimental data on the percentage of free ^103m^Rh radionuclide released from the complexes and eluted from the different columns are summarized in Table 2 and Table 3. There is significant variation in the measured values between columns. This is most likely due to the slight variation in geometry/positioning within the detector between the different individual columns, which impacts the measurement of low-energy gammas.

We evaluated the release of the daughter ^103m^Rh radionuclide from the chelator-bound [^103^Pd]Pd–DOTA-TOC complex using the two types of chromatographic columns. For statistical robustness, four elutions of the [^103^Pd]Pd–DOTA-TOC complex were performed at 24 h intervals over four consecutive days for each of the columns, Strata-X and Strata-C18 (n = 4). The average measured (%) daughter release ratios were 10.5 ± 2.7% from Strata-X and 12.0 ± 0.5% from Strata-C18 with DOTA-TOC, with no significant difference in release rates between them, based on Student’s *t*-test (*p* = 0.282). These findings are consistent with our results for [^103^Pd]Pd–DOTA-TATE, which showed 9.8 ± 3% release from Strata-X, and for [^103^Pd]Pd–phthalocyanine-TATE, which showed 9.6 ± 2.7% release from Strata-X, with no significant difference observed between the two (*p* = 0.829). The absence of statistically significant differences between DOTA- and phthalocyanine-based chelators indicates that π-electron delocalization in the phthalocyanine macrocycle does not provide a measurable protective effect against daughter nuclide release under the studied conditions. The highly localized and intense electronic excitation generated by the Auger electron cascade dominates the release process in both systems, leading to comparable daughter nuclide release behavior. The theoretical Auger electron yield for ^103^Pd decay is reported as ~186% [13]. Together, these results confirm that the ^103m^Rh release ratio is markedly lower than previously predicted based on studies of other Auger electron-emitting radionuclides, including Neodymium-144 (^140^Nd) (88% Auger yield, 95% daughter release) and ^166^Dy (75% Auger yield, 72% daughter release), where we observed strong agreement between the predicted Auger yields and measured daughter dissociation [15,17,27]. This earlier correlation supported the hypothesis that Auger electron cascades create a dense negative electron cloud that destabilizes the daughter ion’s bond to the chelator, consistent with Szilárd’s classical description of radioactive decay altering chemical states rather than recoil-driven dissociation. One possible explanation for this unexpected deviation in the ^103^Pd/^103m^Rh system is that the true Auger electron yield may be closer to ~10% rather than the reported 186%, which is likely a calculated value. This discrepancy suggests inaccuracies in the currently reported nuclear decay data [28], warranting experimental reassessment and direct measurements of the Auger electron yield; however, such work lies beyond the scope of this study.

## 3. Discussion

This study investigated the Szilard–Chalmers effect in the ^103^Pd/^103m^Rh in vivo generator system, where both radionuclides are pure Auger electron emitters with minimal accompanying high-energy radiation. The results are consistent with previous observations for Auger electron-emitting radionuclides, demonstrating that ^103m^Rh daughter isotopes are ejected from their chemical complexation bonds. These findings confirm that the primary mechanism of daughter isotope release is not nuclear recoil, whose kinetic energy is insufficient to break chemical bonds, but rather the localized ionization cascade produced by Auger electrons. This electron cascade creates a transient, negatively charged environment around the decaying atom, destabilizing the coordination bonds and facilitating daughter release, as first conceptualized in Szilard and Chalmers’ early description of nuclear transmutation effects on chemical states [11].

Comparing the effective irradiation time of daughter radionuclides released from an Auger-therapeutic complex with their potential biological effects requires an understanding of ion transport at the molecular level, from the nuclide within a cell, across the cell membrane, and through active and passive transport (diffusion) in the interstitial and extracellular spaces of the body, as discussed in detail in reviews [20,29]. The diffusion of small molecules in the cytoplasm is approximately four times slower than in water, but translational diffusion in cytoplasm still occurs in the millisecond range [30,31]. Ion mobility through ion channels in the cell membrane is also rapid, occurring on the millisecond scale for cations, and this can similarly apply to arsenic-containing ions, which are known to initiate apoptosis [32,33].

The chemical properties of these released radionuclide daughters also influence their radiation risk. The half-lives of these radionuclides typically range from hours to days [34], meaning that, once released, they will decay within the body. As these radionuclides lack preferential organ-specific accumulation, they often bind to proteins in circulation, and, as a result, their radiation dose is primarily delivered to the circulating blood, lymphatic fluids, and clearance organs such as the liver and kidneys. This highlights the need for the careful evaluation of daughter radionuclide redistribution when designing Auger-based therapeutic agents. These risks are, at this stage, theoretical and warrant in vitro cell uptake or membrane transport studies to prove the effect though well-designed future radiobiology studies. The average measured release across all systems determined in this study is only ~10% for the ^103^Pd/^103m^Rh in vivo generator system, and, although lower than anticipated and compared to other systems, without these in vitro radiobiology studies, it is too early to determine whether it will result in clinical off-target radiation.

A possible strategy to minimize irradiation from free daughter radionuclides would be to retain them in the targeted cells, where the secondary Auger electrons that they emit could also contribute to therapeutic efficacy. However, our findings show that modifying the chelator’s electronic properties, using ligands with delocalized π-electrons (phthalocyanine-TATE), surprisingly did not reduce daughter release as reported by Nath et al. [19]. Although phthalocyanines are known for their extended π-electron systems, the energy density associated with the Auger electron cascade following the decay of ^103^Pd is highly localized and generates a strong transient negative charge environment. This localized electronic excitation is likely too intense and short-lived to be effectively dissipated or neutralized by π-electron delocalization. Consequently, it seems that Auger electron-induced ionization and electronic rearrangements dominate the release process in both chelator systems, resulting in comparable daughter nuclide release behavior and statistically non-significant differences between DOTA and phthalocyanine chelators. Both DOTA-TATE and π-electron-rich ligands (-TATE) exhibited similar ejection ratios, demonstrating that the ligand electron density seems to be insufficient to dissipate the Auger electron ionization energy. Metal nanoparticles may provide an alternative solution: the dense electron environment in metallic lattices could dissipate the Auger electron energy and stabilize the daughter isotope, as suggested by recent studies [35].

The average measured release across all systems is only ~10% for the ^103^Pd/^103m^Rh in vivo generator system. This contrasts with the 100% release of Francium-221 (^221^Fr) from the ^225^Ac system [10] and the ~30% release of Bismuth-212 (^212^Bi) from the Lead-212 (^212^Pb) system [36]. Both these radionuclides have already entered clinical trials, with ^225^Ac advancing to Phase III (the ACTION trial). This comparison bodes well for the potential future clinical translation of the promising Auger emitter pair ^103^Pd/^103m^Rh. Overall, this work experimentally validates the Szilard–Chalmers effect in a clinically relevant Auger electron emitter and emphasizes the necessity of integrating nuclear decay physics with chemical design to optimize Auger therapy. Furthermore, inconsistencies in published decay data for ^103^Pd/^103m^Rh [28] highlight the importance of verifying and, if needed, updating nuclear datasets with experimentally determined Auger electron yields to improve the dosimetry accuracy and guide therapeutic development.

## 4. Materials and Methods

Rhodium metal foils (Rh, 99.85% purity; thicknesses: 125 μm, 25 μm, and 12 μm) were obtained from Goodfellow Cambridge, Ltd. (Huntingdon, UK). The anion-exchange resin DOWEX^®^ 1X 8 (strong base anion exchanger, chloride form, 200–400 mesh, 20 mL) and analytical-grade reagents, including hydrochloric acid (HCl, 6 M), nitric acid (HNO_3_, 65% *w*/*w*), ammonium hydroxide (NH_4_OH, 28–30% *w*/*w*), ammonium acetate (CH_3_COONH_4_, 0.4 M, pH 5.5), and ethylenediaminetetraacetic acid disodium salt dihydrate (Na_2_EDTA.2H_2_O, 0.01 M), were purchased from Sigma-Aldrich–Merck (St. Louis, MO, USA). Strata-X (polymeric, reverse-phase sorbent, 30 mg/1 mL, particle size 33 μm) and Strata-C18-E (220 mg/6 mL, particle size 55 μm) solid-phase extraction (SPE) columns were sourced from Phenomenex (Torrance, CA, USA). Chelators DOTA-TATE (DOTA-[Tyr^3^]-octreotate) and phthalocyanine-TATE (the standard TATE peptide functionalized with a phthalocyanine moiety serving as a delocalized π-electron-rich chelator) were custom-synthesized by GL Biochem (Shanghai, China) at 98% purity, and DOTA-TOC (DOTA-[Tyr3]-octreotide) was provided by Pharm-Sintez (Moscow, Russia). All solutions were prepared using deionized water (Milli-Q purification system).

The irradiation of rhodium foils in two separate batches was performed using an MGC-20 cyclotron at ATOMKI. Radioactive rhodium measurements were performed using an Atomlab™ 500 dose calibrator (Mirion Technologies, formerly Biodex, New York City, NY, USA) and a high-purity germanium (HPGe) detector (Canberra, model 2002 CSL, USA). Gamma spectra were analyzed with the Genie-2000 software package. A Thermomixer^®^ C (Eppendorf SE, Hamburg, Germany) was used for mixing during chemical processing steps. All chemical separation and experimental procedures for the first batch were carried out at Necsa, while the second batch was processed at ATOMKI.

### 4.1. Production of ^103^Pd via the Proton Irradiation of ^103^Rh

^103^Pd was produced via the ^103^Rh(p,n)^103^Pd nuclear reaction by irradiating rhodium metal foils with 11 ± 0.05 MeV protons using an MGC-20 compact multiparticle cyclotron at the ATOMKI Institute for Nuclear Research (Debrecen, Hungary). The foils were exposed to a 20 μA proton beam for 30 h, yielding approximately 6 GBq of ^103^Pd activity.

Our cyclotron is equipped with a newly developed and unique beam diagnostic system, namely a compact beam energy monitor (BEM). This device is integrated into the main beam transport channel, enabling the continuous and real-time monitoring of the proton beam energy across all irradiation lines, including the isotope production beamline. The measurement accuracy and performance validation of the BEM have been previously reported and described [37].

### 4.2. Separation of ^103^Pd from Irradiated ^103^Rh

The separation of ^103^Pd ions from the irradiated rhodium targets was performed in two batches, employing two different experimental procedures. In the first batch, the irradiated rhodium foil was transferred to the Radiochemistry Laboratory of the South African Nuclear Energy Corporation (Necsa, Pelindaba, South Africa) for chemical processing. Dissolution of the irradiated target to recover ^103^Pd was achieved by a modified version of the electro-dissolution method described by Lagunas-Solar et al. [38].

Briefly, the rhodium foil was mounted approximately 15 mm under a graphite rod electrode and placed inside a glassy carbon crucible positioned on a copper plate electrode. The two electrodes were connected to a 220 V AC autotransformer. The crucible was filled with concentrated HCl (30 mL) to ensure contact of the liquid with the graphite electrode, and an alternating current of 2–2.5 A was applied across the system. After approximately 2 h, the liquid level in the crucible had dropped due to HCL gas evolution and it was no longer in contact with the graphite electrode, thereby halting the current flow and the dissolution process. The remaining red-colored HCl solution was collected for further purification, and fresh HCl (32%, 30 mL) was added to start the dissolution process again. The procedure was repeated three times until only fragments of the target foil remained, which could no longer maintain contact with the electrodes, thereby preventing further dissolution.

Safety Precautions: The process was conducted in a well-ventilated lab inside a fume hood to safely remove chlorine and hydrogen gases generated during electrolysis, as well as to minimize exposure to evaporating HCl resulting from the highly exothermic electrochemical reaction. The extracted air then passed through a sodium hydroxide scrubber system to neutralize the gas. Essential protective equipment for the process was safety glasses and gloves, with possible use of a half-face mask.

In the second batch, separation of the irradiated rhodium foil was performed using custom-built radionuclide separation equipment (RSE) developed by our group [39]. This system employs a dry distillation technique, whereby ^103^Pd ions are evaporated from ^103^Rh foil and subsequently collected on a designated substrate.

### 4.3. Recovery and Purification of ^103^Pd Activity

^103^Pd activity was recovered from the rhodium solution obtained after dissolution using anion-exchange chromatography, following the method described by Chunfu et al. [40]. A DOWEX 1X8 (Cl^−^/200–400 mesh) anion-exchange column (1.7 × 10 cm, ~20 mL) was prepared and conditioned with 6 M HCl. The Rh/Pd aliquots from dissolution were loaded onto the column, and [^103^Rh]RhCl_6_^3−^ was eluted with 6 M HCl (~60 mL) at a flow rate of approximately 1 mL/min. Under these conditions, the resin selectively retains the ^103^Pd ions. The column was then washed with deionized water (~60 mL) to remove residual chloride. ^103^Pd was subsequently eluted with 6 M NH_4_OH (~50 mL), yielding [^103^Pd]Pd(NH_3_)_4_^2+^ fractions. These fractions were dried under a gentle stream of argon at 40 °C and re-dissolved in 0.1 M NH_4_OAc (pH 5.5). Radioactivity measurements of the purified fractions were performed using a high-purity germanium gamma detector (Canberra GC2518, 24% relative efficiency; Mirion Technologies, Meridan, CT, USA). The radionuclidic purity (along with equilibration to ^103m^Rh daughter radionuclide) was determined as >99%.

The ^103^Pd activity collected on the substrate from the second batch was recovered by acidic treatment. The surface was washed with 2 mL of HCl, and the resulting solution was passed through an anion-exchange resin for purification [39]. The column was subsequently washed with hydrochloric acid and water to remove impurities, and purified ^103^Pd activity was eluted with 1 mL of ammonium hydroxide.

### 4.4. Preparation of ^103^Pd-Labeled Chelator Complexes

The purified ^103^Pd radionuclide was complexed with the strong peptide–chelator bioconjugate DOTA-TATE, dissolved in 0.1 M ammonium acetate buffer (pH 4 to 4.5), by incubation for 1 h at 90 °C. The resulting [^103^Pd]Pd–DOTA-TATE complex was immobilized on the stationary phase of a Strata-X SPE reverse-phase column, which was subsequently washed with 0.01 M EDTA multiple times to remove any unbound chelating agent or free metal ions. As a result, only the chelator-bound ^103^Pd–complex remained retained on the column. The elution and washing procedure itself served as an effective quality control step. The solid-phase extraction columns used were selective in retaining the chelator-bound complexes; therefore, only Pd complexes stably coordinated to the chelator remain immobilized on the column. Any unbound or weakly bound Pd species would have been removed during the washing steps and detected in the eluates. Consequently, the activity measured on the column after washing corresponded exclusively to chelator-complexed Pd, confirming the chemical identity and stability of the Pd complexes under the applied experimental conditions. The complexation of the ^103^Pd radionuclide with phthalocyanine-TATE was performed using the same method, pH, and concentration as for [^103^Pd]Pd–DOTA-TATE complexation. The pH values for the coordination of Pd(II) to phthalocyanines and porphyrins are known to be between 4 and 5.

**Figure 5:**
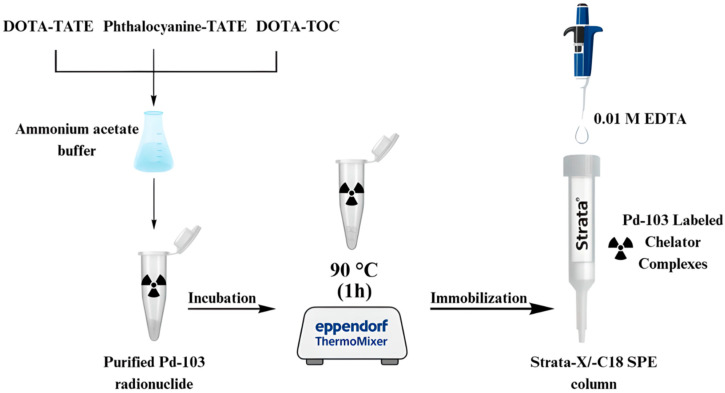
Experimental protocol for the preparation of ^103^Pd-labeled chelator complexes.

The purified ^103^Pd radionuclide obtained from the separation of the second batch was complexed with 10^−3^ M DOTA-TOC chelator, dissolved in 0.4 M ammonium acetate buffer (pH 5.5), by incubation in a thermomixer for 1 h at 90 °C. The resulting [^103^Pd]Pd–DOTA-TOC complex was immobilized onto the stationary phase of a Strata-X SPE reverse-phase column and a Strata-C18 hydrophobic column. The columns were then washed repeatedly with 0.01 M EDTA to remove unbound chelating agents or free metal ions, ensuring that only the chelator-bound ^103^Pd–complex remained retained. The overall preparation procedure is illustrated in Figure 5.

### 4.5. Release of ^103m^Rh Daughter Nuclides from ^103^Pd Complexes

The [^103^Pd]Pd–DOTA-TATE-loaded Strata-X column was eluted with 1 mL of 0.01 M EDTA at 5 h intervals, and the procedure was repeated five times to enable the statistical evaluation of ^103m^Rh radionuclide release. The experiment was subsequently repeated under identical conditions using phthalocyanine-TATE as the chelating agent to investigate the potential protective effect of delocalized π-electrons on daughter nuclide retention. The [^103^Pd]Pd–DOTA-TOC-loaded Strata-X column was eluted daily with 0.8 mL of 0.01 M EDTA over four consecutive days at 24 h intervals. In parallel, the [^103^Pd]Pd–DOTA-TOC-loaded Strata-C18 column was eluted daily with 5 mL of 0.01 M EDTA for the same duration and at the same intervals. The larger elution volume for the Strata-C18 column reflected its approximately fourfold greater packing size compared to the Strata-X column. Radioactivity in both the Strata-X and Strata-C18 columns was measured before and after each elution, and, in the eluates, gamma spectroscopy was used to quantify radionuclide release. The C-18 columns used in the study exclusively bind peptides only and not EDTA or free metal ions. This means that no EDTA is retained on the column. EDTA complexes have very low kinetic inertness and high thermodynamic stability with metal ions, so it is possible to completely complex the free metal ions and wash them out from the column. The EDTA (in excess) removes all unbound metal ions that are due to the after-effect on the column; hence, the activity on the column is measured as it represents the ‘loss’ of the daughter radionuclide.

The eluted activity was attributed to the release of free ^103m^Rh daughter nuclides from the chelator as a consequence of Auger electron-induced ionization. The ^103m^Rh isotope was identified and characterized by its diagnostic X-ray emission at 39.76 keV (0.068% intensity). Gamma emissions were measured using a Canberra HPGe detector (Mirion Technologies Inc., Atlanta, GA, USA), and spectra were analyzed with the Genie 2000 software, V3.4.1. 2016. Decay corrections for activity eluted and remaining on the column as compared to the start activity when the column was prepared were implemented.

**Figure 6:**
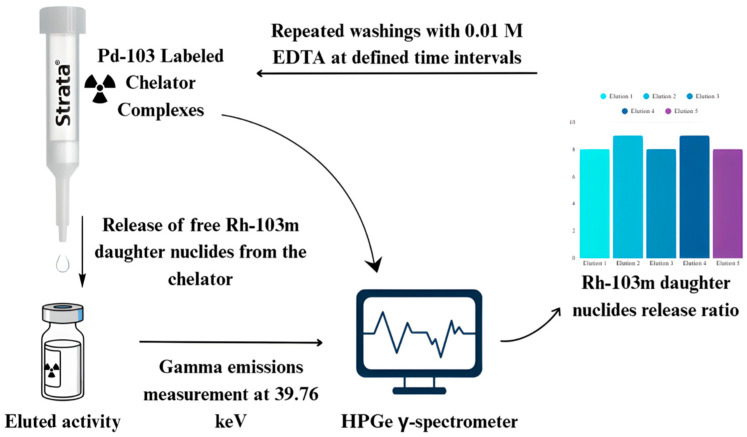
Quantification procedure for ^103m^Rh daughter nuclides released from ^103^Pd–chelator complexes.

Due to the weak intensity of the X-ray, absolute activity quantification was not feasible, as self-absorption effects occurred and the measurement geometry (liquid sample volumes in milliliters rather than point sources) was not optimal. However, since the study focused on activity ratios, the use of identical glassware, equipment, geometry, and measuring times ensured reliable relative measurements. The quantification procedure for evaluating ^103m^Rh release is shown in Figure 6.

## 5. Conclusions

This study investigated the release dynamics of daughter radionuclides from the ^103^Pd/^103m^Rh in vivo generator system via the Szilárd–Chalmers effect. Methodological advances for the evaluation of ^103^Pd-radiolabeled compounds included a high-yield dry distillation process (72–91%) for ^103^Pd separation and an acidic treatment technique achieving 81–83% recovery. The ^103^Pd/^103m^Rh in vivo generator system demonstrated the measurable release of ^103m^Rh from chelator-bound ^103^Pd complexes. The mean ejection/release rates were 9.8 ± 3% from Strata-X with DOTA-TATE; 10.5 ± 2.7% and 12.0 ± 0.5% from Strata-X and Strata-C18 with DOTA-TOC, respectively; and 9.6 ± 2.7% from Strata-X with phthalocyanine-TATE. For the ^103^Pd/^103m^Rh in vivo generator system, only ~10% daughter release was found across all systems, which is significantly lower than for other clinically investigated systems such as ^225^Ac and ^212^Pb. This represents a positive indication for the potential clinical translation of this exciting Auger emitter pair. Furthermore, the use of a π-electron-delocalized chelator (phthalocyanine) did not mitigate this effect, contrary to earlier reports. These values are markedly lower than the theoretical Auger electron emission probability (~186%). One explanation for this difference could be possible inaccuracies in the current decay data, which warrant further investigation to determine if the emission probability is indeed incorrect or the after-effect theory needs further exploration. The findings highlight that Auger electron decay can destabilize daughter radionuclides, creating risks of systemic redistribution despite effective chelation, particularly in rapidly diffusing biological environments. Overall, these results emphasize the need for more accurate nuclear decay data and improved carrier strategies, such as nanoparticle-based delivery systems, to fully exploit the therapeutic potential of Auger emitters while minimizing unintended dose deposition.

## Figures and Tables

**Table 1:** Post-irradiation γ-spectral analysis of ^103^Pd/^103m^Rh and evaluation of potential Rh radionuclidic contaminants produced at 11 MeV proton irradiation.

^103^Pd/^103m^Rh Decay Products	Energy (keV)	Intensity (%)	Identified
	39.7	0.0683	Yes (Evaluated)
	53.3	3 × 10^−5^	No
	62.4	0.00104	Yes
	241.9	5 × 10^−7^	No
	295.0	0.00280	Yes
	317.7	1.50 × 10^−5^	No
	357.5	0.0221	Yes (Evaluated)
	443.8	1.50 × 10^−5^	No
	497.1	0.00396	Yes
Possible ^102^Rh/^102m^Rh contaminants	475.1	46	No
	475.1	95	No
Possible ^101^Rh/^101m^Rh contaminants	198.0	73	No
	306.9	81	No

**Table 2:** Eluted ^103m^Rh (%) from [^103^Pd]Pd–DOTA-TATE (DT)- and [^103^Pd]Pd– phthalocyanine-TATE (P)-loaded Strata-X columns (n = 5), indicating recoil-induced dissociation from chelator-bound complexes.

Elution Cycle	Eluted ^103m^Rh (%) from the Strata-X Column (n = 5)	Average Eluted ^103m^Rh (%)
DT/1	8.1 ± 1.6	9.8 ± 3
DT/2	9.4 ± 2.2	
DT/3	8.9 ± 3.2	
DT/4	10.4 ± 4.0	
DT/5	12.1 ± 4.0	
P/1	10.1 ± 2.9	9.6 ± 2.7
P/2	8.7 ± 2.7	
P/3	10.0 ± 2.4	

Data represent mean ± SD (standard deviation); n = number of elutions per column.

**Table 3:** Eluted ^103m^Rh (%) from [^103^Pd]Pd–DOTA-TOC (DC)-loaded Strata-X (n = 4) and Strata-C18 (n = 4) columns, indicating recoil-induced dissociation from chelator-bound complexes.

Elution Cycle	Eluted ^103m^Rh (%) from the Strata-X Column (n = 4)	Eluted ^103m^Rh (%) from the Strata-C18 Column (n = 4)
DC/1	5.0	12.3
DC/2	12.8	12.3
DC/3	10.6	11.0
DC/4	13.4	12.4
Average Eluted ^103m^Rh (%)	10.5 ± 2.7	12.0 ± 0.5

## Data Availability

The original contributions presented in the study are included in the article, further inquiries can be directed to the corresponding author.

## Abbreviations

The following abbreviations are used in this manuscript:

SPE	Solid-phase extraction
TRNT	Targeted radionuclide therapy
β−	Beta (minus) particle/emitter
α	Alpha particle/emitter
DSB(s)	Double-strand break(s)
LET	Linear energy transfer
DOTA	1,4,7,10-Tetraazacyclododecane-1,4,7,10-tetraacetic acid
PRRT	Peptide receptor radionuclide therapy
EOB	End of bombardment
RSE	Radionuclide separation equipment
HPGe	High-purity germanium
EDTA	Ethylenediaminetetraacetic acid
SD	Standard deviation
γ	Gamma radiation
